# Role of Acidic Residues in Helices TH8–TH9 in Membrane Interactions of the Diphtheria Toxin T Domain

**DOI:** 10.3390/toxins7041303

**Published:** 2015-04-14

**Authors:** Chiranjib Ghatak, Mykola V. Rodnin, Mauricio Vargas-Uribe, Andrew J. McCluskey, Jose C. Flores-Canales, Maria Kurnikova, Alexey S. Ladokhin

**Affiliations:** 1 Department of Biochemistry and Molecular Biology, University of Kansas Medical Center, Kansas City, KS 66160, USA; E-Mails: c.ghatak79@gmail.com (C.G.); mrodnin@kumc.edu (M.V.R.); mvargas@kumc.edu (M.V.-U.); 2 Department of Microbiology and Immunobiology, Harvard Medical School, Boston, MA 02115, USA; E-Mail: andrew_mccluskey@hms.harvard.edu; 3 Department of Chemistry, Carnegie Mellon University, Pittsburgh, PA 15213, USA; E-Mails: jose.florescanales@gmail.com (J.C.F.-C.); kurnikova@cmu.edu (M.K.)

**Keywords:** pH-trigger, conformational switching, membrane insertion

## Abstract

The pH-triggered membrane insertion of the diphtheria toxin translocation domain (T domain) results in transferring the catalytic domain into the cytosol, which is relevant to potential biomedical applications as a cargo-delivery system. Protonation of residues is suggested to play a key role in the process, and residues E349, D352 and E362 are of particular interest because of their location within the membrane insertion unit TH8–TH9. We have used various spectroscopic, computational and functional assays to characterize the properties of the T domain carrying the double mutation E349Q/D352N or the single mutation E362Q. Vesicle leakage measurements indicate that both mutants interact with the membrane under less acidic conditions than the wild-type. Thermal unfolding and fluorescence measurements, complemented with molecular dynamics simulations, suggest that the mutant E362Q is more susceptible to acid destabilization because of disruption of native intramolecular contacts. Fluorescence experiments show that removal of the charge in E362Q, and not in E349Q/D352N, is important for insertion of TH8–TH9. Both mutants adopt a final functional state upon further acidification. We conclude that these acidic residues are involved in the pH-dependent action of the T domain, and their replacements can be used for fine tuning the pH range of membrane interactions.

## 1. Introduction

Many bacterial toxins, such as diphtheria [1], botulinum [2], tetanus [3] and anthrax [4], rely on environment-induced conformational changes to deliver their active moieties into cells. The diphtheria toxin, for example, delivers its own catalytic domain into the cytosol in response to endosomal acidification. In this case, the translocation domain (T domain) undergoes a series of conformational changes triggered by low pH, resulting in its membrane insertion and the translocation of its *N*-terminus with the attached catalytic domain across the membrane [5]. This remarkable feature is of great importance for potential therapeutic applications based on cellular delivery of cargo molecules into target cells [6,7] and for anti-cancer therapies as a component of immunotoxins [8,9]. However, in order to engineer a cellular delivery platform, it is crucial to decipher the main principles of conformational switching and the molecular details of the pH-induced membrane insertion of the T domain.

**Figure 1:**
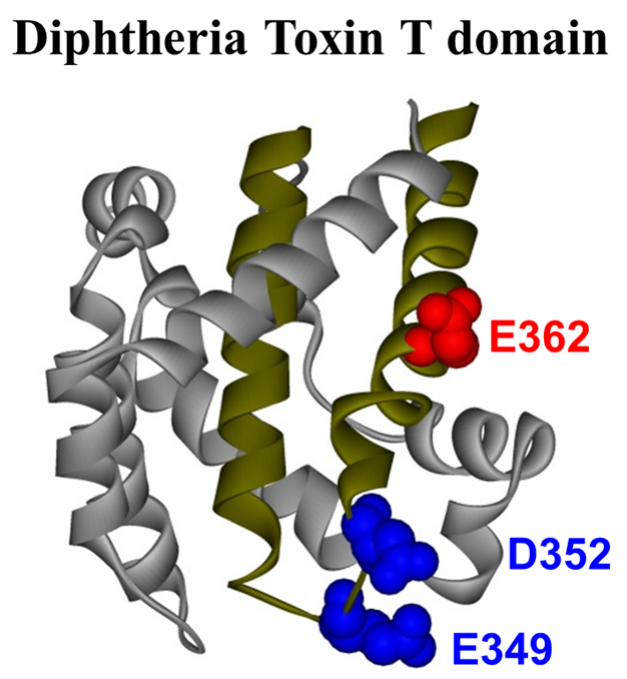
Crystal structure of the diphtheria toxin translocation domain (T domain) in solution at neutral pH [10], highlighting hydrophobic helices TH8 and TH9 as dark yellow ribbons. Acidic residues mutated in this study are shown as CPK representations in blue and red.

The crystal structure of the T domain in solution at neutral pH [10] consists of a globular protein of nine α-helices (named TH1-TH9) of various lengths (see Figure 1), with the most hydrophobic helices, TH8 and TH9 (Figure 1, dark yellow helices), forming the hydrophobic core of the protein. There is no high-resolution structure available for the T domain in its membrane-inserted state, but several studies have demonstrated that helices TH8–TH9 insert as a transmembrane hairpin, while other helices can adopt multiple conformations [11,12,13,14,15,16,17]. Previously, we have characterized the kinetic membrane insertion pathway [18], which comprises a series of conformational transitions occurring first in solution and then in the membrane. We have also determined the free energy stabilizing the different intermediates [19,20] along the pathway.

It is suggested that the protonation of titratable residues (*i.e.*, histidine and glutamic and aspartic acid) plays a key role in different stages along the pathway [21]. We have indeed confirmed that protonation of histidine residues is required for proper conformational changes in solution and in the membrane [21,22,23,24,25]. For example, protonation of residue H257 was found to be critical for triggering the initial conformational change of the T domain in solution, while H322 appears to be important for proper refolding of *N*-terminal helices upon transmembrane insertion of TH8–TH9. However, the role of protonation of acidic residues has been studied to a lesser extent. The T domain has 23 acidic residues, most of which are located in the polar part of the sequence. In this study, we focus on three of them that are located within the hydrophobic consensus membrane insertion unit formed by the TH8–TH9 hairpin, which suggests that they play a direct role in the membrane interactions of the T domain. These residues are E362, which is located in the middle of TH9, and E349 and D352, both located in the loop connecting helices TH8 and TH9 (Figure 1). Although previous mutagenesis studies suggested the importance of these residues in the functionality of the full-length toxin [26,27,28,29], the effect of their replacement on the isolated T domain has not been characterized.

In this work, we replaced these acidic residues with their corresponding amides and characterized the membrane interactions of the resulting mutants using an array of fluorescence-based methods, circular dichroism and molecular dynamics (MD) computer simulations. We generated the double mutant E349Q/D352N (tip of the hairpin) and the single mutant E362Q (middle of TH9) to neutralize the negative charges on the hairpin. We complement our study with the application of *in vitro* and *in vivo* functional assays and describe a model proposing the role of these residues in the T domain’s insertion pathway.

## 2. Results

Since the residues E349, D352 and E362 are located within helices TH8 and TH9 (Figure 1), which form the membrane insertion unit, their protonation may be involved in the pH-induced membrane interactions of the T domain. We have previously combined site-directed mutagenesis with spectroscopic and functional assays to establish the role of six histidines along the membrane insertion pathway of the T domain [22,23,24,25]. Following a similar approach, we generated the double mutant E349Q/D352N and the single mutant E362Q, aimed at removing the negative charges from the tip of the TH8–TH9 hairpin or the middle of TH9, respectively.

**Figure 2:**
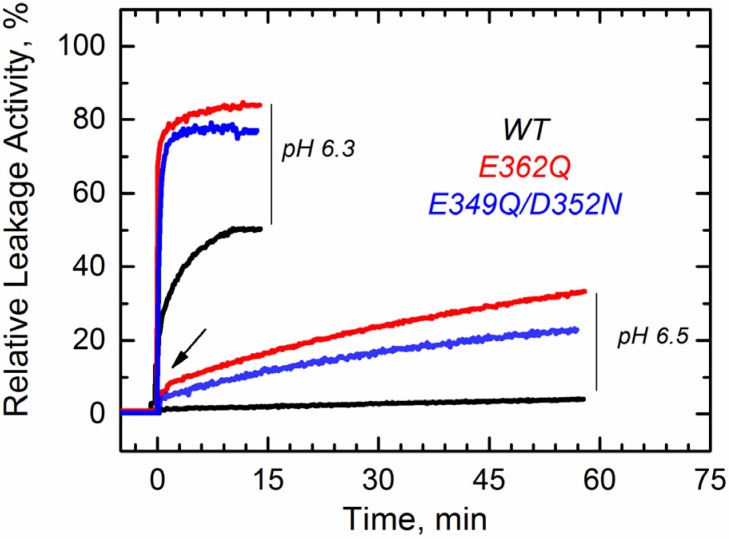
Leakage of lipid vesicle content results from the membrane interactions of the T domain WT (black) and the mutants E349Q/D352N (blue) and E362Q (red). Both mutants appear to perturb the membrane integrity at higher pH than the WT. The arrow indicates the point at which acid was added. Data are representative of at least three independent measurements.

We first tested the ability of these mutants to interact with the membrane using a vesicle permeabilization assay previously applied to characterize membrane interactions of the T domain [22,23]. This leakage assay, although not particularly specific, is a sensitive tool for a rapid assessment of the membrane interaction of the T domain. In the assay, we follow the change in fluorescence intensity associated with the release of the fluorophore/quencher pair). 8-aminionapthalene 1,3,6-trisulfonic acid (ANTX)/p-xylene-bis-pyridinium bromide (DPX) from pre-loaded vesicles induced by the T domain upon acidification of the solution. In Figure 2, we show a typical result of a leakage experiment, where both mutants cause faster and more complete leakage of the markers than the wild-type (WT) at pH 6.3. At pH 6.5, both mutants still induce release of ANTX/DPX, albeit to a lesser extent than at pH 6.3, while the WT does not cause leakage anymore. No leakage was observed at pH 7.5 and above, as judged by the inability of the mutants to cause leakage before the addition of acid (arrow), suggesting that removing the negative charges alone is not sufficient to ensure membrane interactions. These results indicate that both mutants interact with and perturb the membrane at pH values higher than those required for the WT T domain.

**Figure 3:**
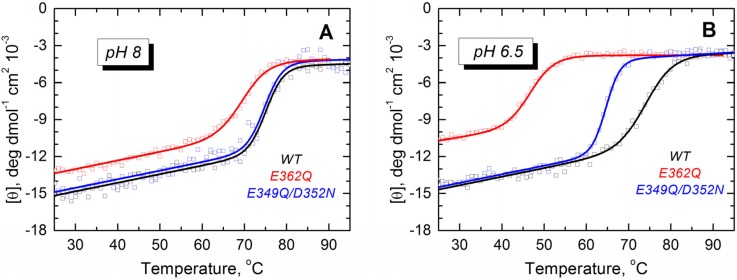
Thermal unfolding measurements of the WT T domain (black) and the mutants E362Q (red) and E349Q/D352N (blue) show that the single mutation causes loss of thermal stability in solution at pH 8.0 (**A**) and 6.5 (**B**). The double mutation affects the stability of the T domain only at the more acidic pH. Measurements were reproduced at least three times for each mutant at each pH.

Next, we characterized the membrane insertion pathway of the mutants using methods developed in previous studies to investigate the T domain WT and mutants [18,22,23,24,25,30]. The first step in the pathway of the WT protein involves a conformational change in solution detectable through changes in the thermodynamic stability [21]. We examined the folding and thermodynamic stability of the mutants in solution using CD spectroscopy. At pH 8 and 6.5, the WT protein and both mutants show CD spectra typical of α-helical proteins (Supplemental Data, Figure S1). However, the molar ellipticity at 222 nm is reduced by 2.0 and 2.5 mdeg·cm^2^/dmol for the mutant E362Q at pH 8 and 6.5, respectively (about 14% and 19% less ellipticity than the WT protein). We studied the thermal stability by monitoring the molar ellipticity at 222 nm as a function of the temperature. The mutant E362Q shows a reduced stability at pH 8 and 6.5 (Figure 3), as evidenced by the shift in the thermal unfolding curves and the quantitative analysis of thermal unfolding (Table 1). The double mutation affects the thermal stability only at pH 6.5, but the changes are not as prominent as those induced by the single mutation. Reliable thermal unfolding measurements are experimentally challenging below pH 6.5, because the T domain tends to aggregate [19,20,31,32]. Nonetheless, our results clearly indicate that the mutant E362Q is less resistant to acid destabilization than the double mutant E349Q/D352N or the WT T domain.

**Table 1:** Thermodynamic parameters of the thermal unfolding of the WT T domain and mutants E362Q and E349Q/D352N in solution.

T domain	pH 8.0		pH 6.5	
	Tm, °C	ΔH°, Kcal/mole	Tm, °C	ΔH°, Kcal/mole
WT	75 ± 1	95 ± 7	74 ± 1	76 ± 6
E349Q/D352N	75 ± 1	113 ± 16	65 ± 1	110 ± 7
E362Q	69 ± 1	73 ± 5	47 ± 1	71 ± 2

**Figure 4:**
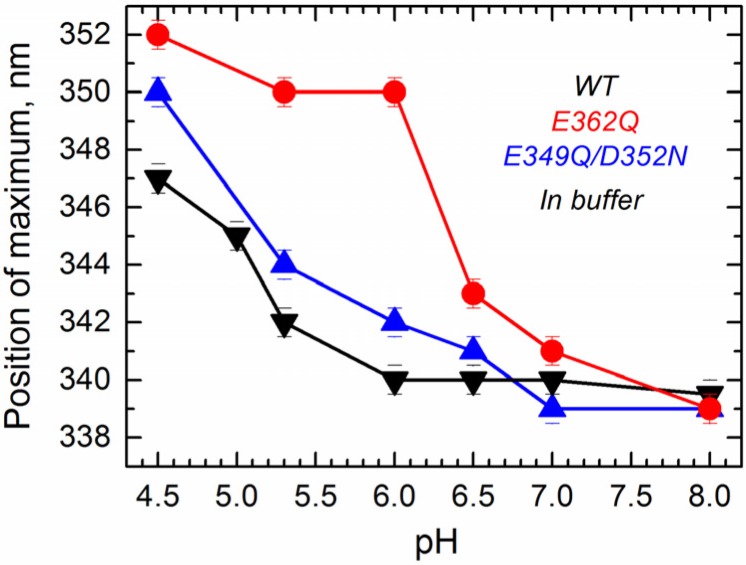
Intrinsic fluorescence measurements in solution show that acidification causes a red-shift in the position of maximum emission in the WT T domain (black) and the mutants E362Q (red) and E349Q/D352N (blue), which is considerable larger for the mutant E362Q. Data correspond to the mean of three independent measurements. Error bars correspond to the instrumental error.

Next, we measured tryptophan fluorescence originating from W206 and W281 located within the *N*-terminal region of the T domain (TH1 and TH5). We have previously shown that the position of maximum emission of tryptophan, which can be reliably obtained by fitting the spectra to a log normal distribution [33], is indicative of conformational changes occurring within the *N*-terminal region of the T domain [24]. Intrinsic fluorescence measurements in solution show that the position of maximum fluorescence emission undergoes a pH-dependent red-shift for all of the proteins (Figure 4), indicating that WT and mutants experience a pH-dependent conformational change in solution. Fluorescence red-shift, normally associated with an increased level of exposure of tryptophan residues to the aqueous phase [33], appears to be significantly larger for the single mutant E362Q than for the WT or the double mutant E349Q/D352N. This result indicates that the mutation E362Q causes a pH-dependent structural change that is distinctively different from that of the WT protein, confirming the results observed with the CD measurements.

**Figure 5:**
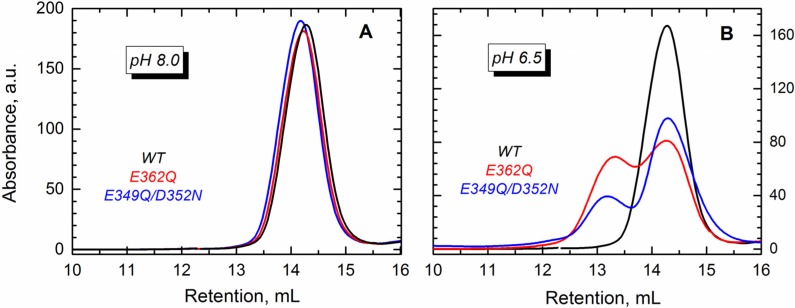
Size exclusion chromatography shows that the WT T domain (black) and the mutants E362Q (red) and E349Q/D352N (blue) elute as a single peak in buffer at pH 8.0 (**A**), which corresponds to the retention volume of the monomeric form of the protein. At pH 6.5 (**B**), both mutants showed an additional peak of earlier elution (~13 mL) that suggests the presence of dimers.

The conformational changes induced by lowering the pH lead to partial exposure of the hydrophobic core of the T domain, which in the absence of membranes, leads to oligomerization [32]. In the WT protein, the dimers start forming at pH < 6 [34], but in mutants with an altered distribution of charges, the dimerization may occur at higher pH. To test this hypothesis, we carried out size exclusion chromatography for the WT and the two mutants. Our results show that either of the proteins eluted as a single peak at pH 8.0 (Figure 5A), which corresponds to the monomeric T domain. The mutants, however, eluted as two peaks at pH 6.5 (Figure 5B), suggesting the formation of dimers.

**Figure 6:**
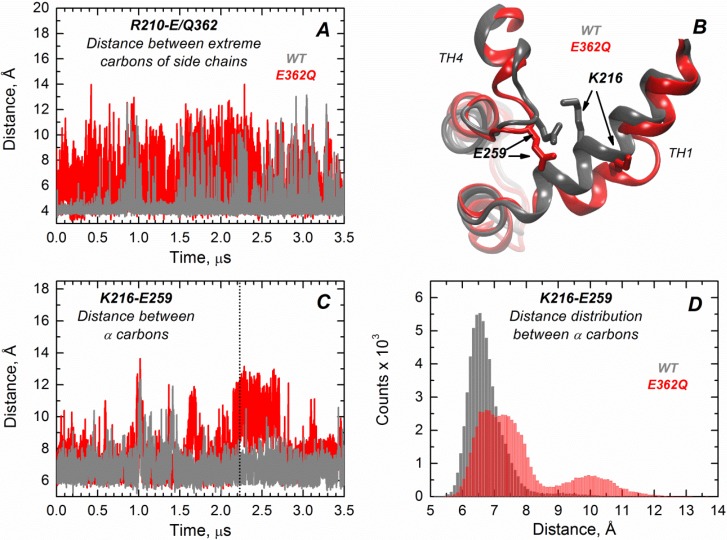
Extended MD simulations of the T domain WT (gray) and mutant E362Q (red) in solution reveal remote structural rearrangement. (**A**) Distance fluctuations between the extreme carbons of side chains of R210 (TH1) and E362 (Q362 in the mutant) are increased in the mutant E362Q, suggesting the loss of a salt bridge between TH1 and TH9; (**B**) Overlap of the structures of the WT and mutant E362Q in the snapshot at 2.2 μs of simulation (dotted line in (**C**)) depicting the structural changes at the interface TH1/TH4 upon mutation. (**C**) Distance fluctuations between α carbons of residues K216 (TH1) and E259 (TH4) are increased upon replacement of E362, suggesting a remote effect of the mutation; (**D**) The distribution of distances between α carbons of residues K216 and E259 (from data of (**C**)) indicates an increased and bimodal distance distribution between both residues in the mutant.

We performed extended molecular dynamics (MD) simulations (3.5 μs) of both the WT T domain and the mutant E362Q in explicit water. This allows us to better visualize the effect of introducing this mutation in the middle of TH9 on the solution folding of the protein. Overall, the results indicate that there is more flexibility in the mutant protein, with occasional bending and partial loss of secondary structure in helix TH1. The latter is consistent with the partial loss of ellipticity observed in CD measurements (Figure S1). Residues of helix TH9 lose contacts with residues of TH1 in the mutant, possibly because of disruption of the salt bridge between R210 and residue 362 upon mutation. Figure 6A illustrates the distance variations between the residue R210 (TH1) and E/Q362 (TH9), where the mutant (red trajectory) shows increased fluctuations in distance compared to the WT protein (grey trajectory) along the entire simulation (notice that the position 362 is a glutamic acid in the WT protein and a glutamine in the mutant protein). Interestingly, TH1 also loses contacts with TH4, suggesting that the mutation in the middle of TH9 induces remote structural rearrangements. Figure 6B displays the structural overlap of the region comprising the TH1/TH4 interface for the WT T domain and the mutant, which illustrates the loss of the salt bridge between residues K216 (TH1) and E259 (TH4) in the mutant. Figure 6C,D shows the distance fluctuations between Cα of residues K216 and E259 along the trajectory, which further indicates the breaking of contacts between TH1 and TH4 in the mutant protein. The disruption of both salt bridges upon mutation is likely to contribute to reduced thermal stability of the mutant E362Q (Figure 3). The interface TH1/TH4 has been shown to play an important role in the formation of the membrane-competent state of the T domain, because histidines H257 and H223 are located in the same interface [21,22,25]. Thus, our MD simulations are consistent with the observed shift in the pH required for the initial conformational changes of E362Q in solution (Figure 2, Figure 3 and Figure 4), with the mutation inducing a structural rearrangement in a remote interface that is important for sensing the pH trigger during the initial conformational switching.

**Figure 7:**
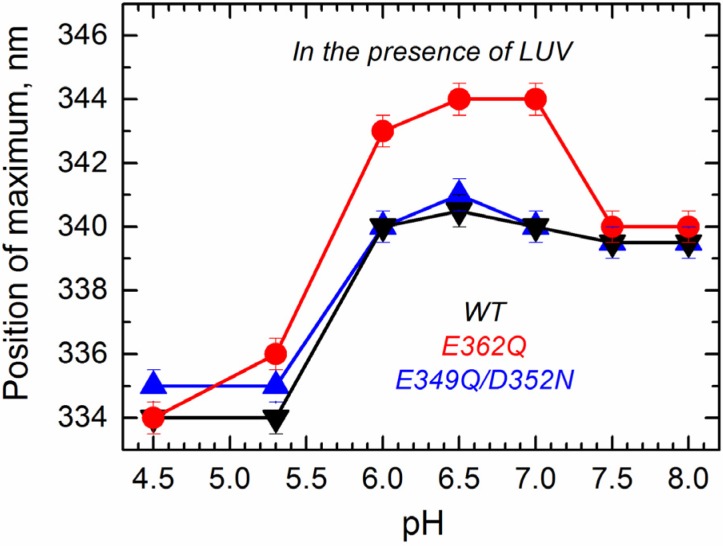
Intrinsic fluorescence measurements in the presence of the bilayer (palmitoyl-oleoyl-phosphatidylglycerol (POPG):palmitoyl-oleoyl-phosphatidylcholine (POPC) 3:1 large unilamellar vesicles (LUV)) show a spectral red-shift for the mutant E362Q (red) at a mildly acidic pH and a spectral blue-shift for the three proteins at a more acidic pH. Values correspond to the position of maximum after averaging five recordings. Error bars are the instrumental error.

To determine how the altered transitions in solution affect the mutants’ folding transitions within the membrane, we applied several methods of fluorescence spectroscopy that allowed us to observe environment-induced spectral changes of intrinsic and extrinsic probes. First, we measured the spectral position of tryptophan fluorescence of the WT and the mutants in the presence of large unilamellar vesicles (LUV) composed of palmitoyl-oleoyl-phosphatidylglycerol (POPG) and palmitoyl-oleoyl-phosphatidylcholine (POPC) in a 3:1 ratio as a function of pH. The data presented in Figure 7 show that the E349Q/D352N mutant behaves similarly as the WT T domain, showing little spectral changes above pH 6.0 and a strong blue shift below pH 5.3. In contrast, the E362Q mutant exhibits a red shift of fluorescence (pH 6.0–7.0) before showing the blue shift below pH 5.3. Note that spectral positions for this mutant under mildly acidic conditions are different than those observed in buffer (compare the red curves in Figure 4 and Figure 7), indicating that the red shift results from a protein conformation adopted in the membrane. A similar spectroscopic signature has been previously observed in mutants with replacements of the *C*-terminal histidines [24], where the red shift correlated with misfolding of the *N*-terminal region in the membrane (see the Discussion). The fact that further acidification results in a blue-shifted tryptophan spectrum suggests that E362Q adopts a WT-like conformation regardless of its early misfolding in the membrane.

**Figure 8:**
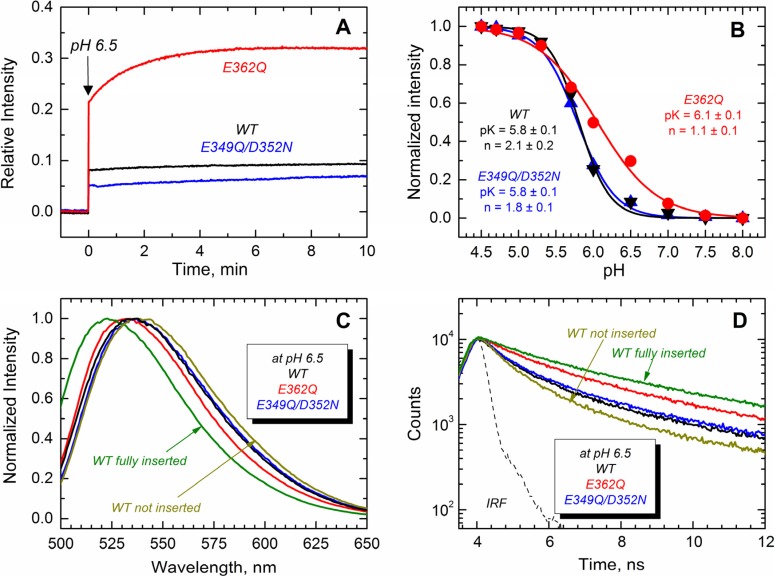
Transmembrane insertion of the TH8–TH9 hairpin into 75POPG/25POPC LUV measured at different pHs for the T domain WT (black) and the mutants E362Q (red) and E349Q/D352N (blue). (**A**) Kinetics of insertion of the NBD-labeled T domain at residue N366C showing a more efficient insertion of the mutant E362Q at pH 6.5; (**B**) Normalized NBD intensity at 523 nm for the T domain WT and mutant upon mixture with LUV and equilibration at the indicated pH. The apparent *pK* and the cooperativity factor *n* were estimated by fitting the data as previously described [35]; (**C**) Normalized emission spectra of the data of (**A**) at the end of the kinetics showing a blue-shift in the position of maximum emission for the mutant E362Q (λ_max_ of the spectra are 536 nm, 536 nm and 531 nm for WT, E349Q/D352N and E362Q, respectively; λ_max_ of reference spectra are 536 nm and 523 nm for the non-inserted and fully-inserted WT T domain); (**D**) Fluorescence lifetime of the data of (**A**) at the end of the kinetics of insertion showing longer fluorescence decay for the mutant E362Q (average τ values for decays are 1.5 ns, 1.7 ns and 2.5 ns for WT, E349Q/D352N and E362Q, respectively; average τ values for reference decays are 1.2 ns and 3.3 ns for the non-inserted and fully-inserted WT T domain, respectively). The instrument response function (IRF) was used for the deconvolution of the data. Data for a non-inserted and fully-inserted WT T domain were measured at pH 8.0 and pH 5.3, respectively.

Next, we monitored the transbilayer insertion of the TH8–TH9 hairpin using the environment-sensitive NBD fluorophore specifically attached in the middle of what becomes a transmembrane region [18,23]. To achieve site-selective labeling with NBD, a unique cysteine residue at position 366 (middle of TH9) was introduced in all of the proteins. The results displayed in Figure 8A show that only the E362Q mutant exhibits an increase of NBD emission intensity in the presence of LUV at pH 6.5, while all of the proteins show a more substantial increase of the emission intensity at pH 5.3 (Figure 8B). All of the changes in fluorescence intensity are accompanied by blue-shifted spectra and longer fluorescence decay, which is consistent with the NBD probe being relocated into the more hydrophobic environment of the membrane hydrocarbon core. Panels C and D show the spectral changes associated with the membrane interactions of the proteins at pH 6.5, where the E362Q mutant exhibits a 5-nm blue-shift and a 1-ns lengthening of the life time. The spectral properties of the WT T domain in the presence of LUV at pH 8.0 and 5.3 are shown for comparison as non-inserted and fully-inserted populations, respectively. Panel B shows the relative changes in NBD fluorescence upon equilibration at the indicated pH. We estimated the apparent *pK* of membrane insertion and the cooperativity factor by fitting the data as described previously [35]. While the double mutation E349Q/D352N affects none of these thermodynamic parameters, the mutation E362Q increases the *pK* by 0.3 points and decreases the cooperativity from about 2 to ~1. The latter suggests that residue E362 is protonated during the membrane insertion process (see the Discussion). Our results thus indicate that both mutants are able to adopt a transmembrane topology, but the mutant E362Q does it at a higher pH than that required for the WT and the double mutant E349Q/D352N.

**Figure 9:**
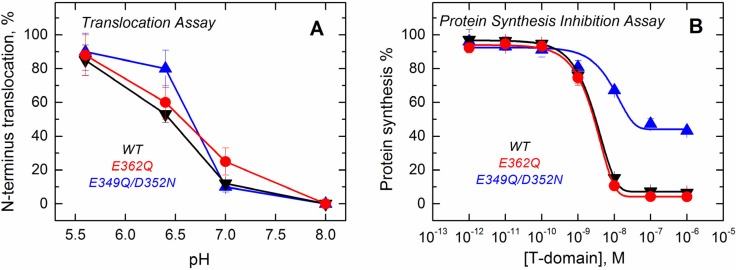
Functional assays to study the activity of the T domain WT (black) and the mutants E362Q (red) and E349Q/D352N (blue). (**A**) The translocation activity of the isolated T domain, carried out by using a proteolytic assay that cleaves the *N*-terminus when translocated to the lumen of thrombin-loaded vesicles, indicates that both of the mutants have a level of activity similar to that of the WT; (**B**) The cell death assay with the entire toxin, performed using the protein synthesis inhibition assay by measuring the [^3^H]-leucine incorporation in treated cells and normalized against untreated cells, shows that only the double mutant E349Q/D352N loses part of its activity. Each point is the average of four replicates. Error bars correspond to the standard deviation.

Since the formation of the final functional state of the T domain requires the translocation of its own *N*-terminus, we assessed the ability of the mutants to perform this action using a translocation assay described previously [36]. The assay is based on proteolytic cleavage of a His-tag attached to the *N*-terminus of the T domain with a thrombin cleavage site. When the *N*-terminus of the T domain is translocated into the lumen of a thrombin pre-loaded vesicle, the protease cleaves off the His-tag, changing the electrophoretic mobility of the protein (Supplemental Data, Figure S3). The results presented in Figure 9A show that the mutants retain the ability to translocate their *N*-terminus across the bilayer and that the pH dependence of translocation observed for the mutants is similar to that of the WT.

Finally, we tested the effect of these mutations on the ability of the T domain to translocate the catalytic domain into cells in the context of the full-length diphtheria toxin. Since the catalytic domain must be translocated into the cytosol to inhibit protein synthesis, measuring the incorporation of radiolabeled leucine by living cells is an accepted test of the functionality of the T domain [37]. The results displayed in Figure 9B show that the mutation E362Q does not lead to any detectable changes in the inhibitory potency of the diphtheria toxin over protein synthesis. The full-length toxin carrying the mutation E349Q/D352N, on the other hand, appears to be only half as efficient in inhibiting protein synthesis. In our previous studies, we encountered several T domain mutants with altered translocating abilities; however, in those cases, sufficiently high concentrations of protein would result in low synthesis, and the inhibition curves just appear to shift to higher protein concentrations [22,23]. This, apparently, is not the case for the E349Q/D352N mutant, and we suspect that these replacements result in some nonproductive interactions, which are not related to the T domain’s membrane insertion. Interestingly, the E362Q mutant, which showed the biggest changes in solution folding and membrane interactions in the context of the isolated T domain, is fully active in the context of the full-length toxin.

## 3. Discussion and Conclusions

The membrane insertion of the diphtheria toxin T domain is a complex refolding process that occurs in response to the acidification of the medium [21]. It has been suggested that the protonation of key titratable residues triggers and controls a set of conformational changes (occurring in solution and in the membrane) that brings the T domain to its final functional state [21,22,23,24,25,30,38]. We have previously combined site-directed mutagenesis with spectroscopic and functional assays to establish the role of the six histidine residues in triggering the initial conformational changes in solution [22,25] and regulating the late stages of refolding within the bilayer [23,24]. Here, we used a similar approach to study the role of three acidic residues (E349, D352 and E362), located within the transmembrane insertion unit TH8–TH9 (Figure 1), and we found that their replacement with their corresponding amide derivatives results in changes in the membrane interactions of the T domain.

Since the titratable residues E349, D352 and E362 are located within the membrane insertion unit TH8–TH9 (Figure 1), their protonation is expected to affect the pH-induced membrane interactions of the T domain. Indeed, previous studies have shown loss of activity of the full-length diphtheria toxin in living cells upon replacement of E349 and D352 [26,27]. The overall effects of replacing these residues with either alanine or corresponding amides appear to be modest, while introduction of the positive charge in the tip of TH8–TH9 led to complete loss of activity. In either study, no detailed characterization of how these mutations affect the early stages of the insertion pathway was presented, making it difficult to decipher the effects of possible misfolding in solution and on the membrane interface from those of aberrant transmembrane insertion. (e.g., we found that double replacement E349K/D352K results in misfolding of the T domain in solution (Supplemental Data, Figure S2)). To the best of our knowledge, until now, no systematic studies have been carried out for mutants replacing E362 either.

**Figure 10:**
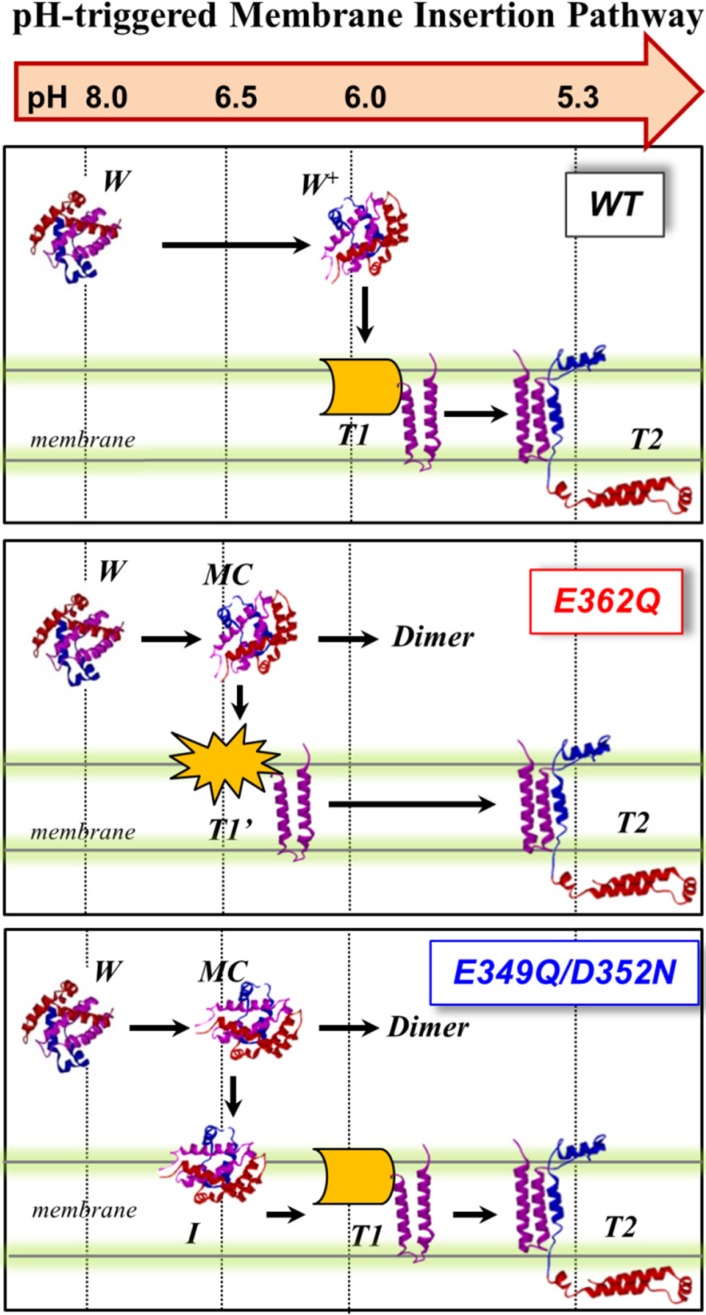
pH-dependent membrane insertion pathway of the T domain WT and the mutants E362Q and E49Q/D352N. For the WT, the pathway consists of an initial conformational change in solution (membrane-incompetent W-state to membrane-competent W^+^-state), followed by association with the membrane and transbilayer insertion into the T-states. The T1- and T2- states are states with the *N*-terminus located on the *cis* and *trans* side of the bilayer, respectively. For both mutants, the formation of a membrane-competent state (MC-state) is shifted towards less acidic conditions. Without a membrane, aggregation occurs under less acidic conditions than the WT. With a membrane, E362Q readily inserts into the bilayer, while the double mutant accumulates interfacially (I-state). The *N*-terminal region of E362Q misfolds within the membrane interface; nevertheless, additional acidification results in the completion of the pathway and formation of the functional inserted state.

We summarize our current results obtained with the two mutants and those previously published with the WT in Figure 10, which proposes a schematic interpretation of the effect of the mutations on the membrane insertion pathway of the T domain. In the WT protein (top panel), the T domain initially undergoes a pH-triggered conformational change brought about by the protonation of key histidine residues in solution [22,25]. The resulting protonated membrane-competent state (W^+^-state) associates with the membrane interface, where depending on the lipid composition, it can either get trapped in the nonproductive interfacial intermediate I-state or proceed along the pathway by inserting the TH8–TH9 helical hairpin across the lipid bilayer (T1-state). In this study, we used a lipid composition with a high fraction of anionic lipids, which lowers the kinetic barrier for insertion and assures that there is no accumulation of the interfacial I-state [18,19]. The T1-state, in which the *N*-terminal region still exists on the *cis* side of the bilayer, is subsequently converted into the T2-state (the so-called open-channel state [14]), with the *N*-terminus accessible to the *trans* side of the bilayer.

The mutations E362Q and E349D/D352N caused changes in the membrane insertion pathway of the T domain, especially in the initial stages (Figure 10, middle and bottom panel). In the scheme, the pH range for the formation of the membrane-competent state (MC-state) of the mutants is shown to be shifted towards less acidic conditions, when compared to the WT protein, because both mutants are more efficient than the WT T domain at permeabilizing vesicles (Figure 2). The ANTS/DPX-based vesicle leakage assay, however, does not discriminate transient interfacial interaction of the T domain from the formation of a stable transmembrane structure, as both can result in vesicle permeabilization (e.g., as discussed in [23]). Indeed, the two mutants adopt different topologies when initially partitioning to the membrane (see the discussion below). It is likely that both mutants bypass the conventional conformational switching of the WT T domain, which requires a specific pH range to protonate the key residues triggering the initial conformational change [25]. Nevertheless, acidification is still needed to trigger the initial conformational changes, which indicates that the alternative pathway still utilizes the protonation of histidine residues. The greater loss of thermal stability upon acidification (Figure 3) and the larger red-shift in the intrinsic fluorescence measurements (Figure 4) support the idea of an alternative pathway for the formation of the membrane-competent state in the case of the mutants.

While both mutants interact with the membrane at pH 6.5, only E362Q can readily adopt the transmembrane T1-state at this pH (Figure 8A,C,D). Both the WT T domain and the double mutant E349Q/D352N exhibit identical pH dependence of the transmembrane insertion of TH8–9 (Figure 8B) with an apparent *pK* of 5.8 and cooperativity factor *n* ~ 2. The latter can be interpreted as the requirement for two titratable residues to be protonated during the insertion. We suggest that one of these residues is E362, because its replacement leads to a shift of *pK* to 6.1 and a decrease of cooperativity *n* ~ 1 (another residue that requires protonation is likely to be H257, acting as a main destabilizer of the solution fold [22,25]). Thus, E362Q mutation mimics the protonation that removes insertion-blocking charge from the middle of TH9, resulting in the mutant inserting into the bilayer under less acidic conditions and with a decreased cooperativity. The double mutant still needs the protonation of E362 and consequently adopts the T1-state at the same pH range as the WT T domain.

In the WT T domain, the insertion of the TH8–TH9 hairpin can be uncoupled from the insertion of the *N*-terminal helices and the translocation of the *N*-terminus by mutating out the histidines flanking TH8–TH9 sequence [23,24], producing partially misfolded and inactive mutants. The spectral behavior of the mutant E362Q (red shifted spectra of Figure 7) resembles the misfolding of the *N*-terminal helices within the membrane observed in the histidine mutants. Unlike the case of the histidine mutants, however, the further acidification corrects the misfolding, and E362Q adopts a functional T2-state (Figure 7 and Figure 9). Thus, the shift in in pH-dependence of the insertion of the TH8–TH9 hairpin does not result in a corresponding shift of the insertion of *N*-terminal helices of the T domain. The latter clearly requires protonation of other titratable residues located outside of the TH8–TH9 hairpin. There are two main candidates, with one being H322, which are critical for the formation of the T2 state [24]. Another possibility is that protonation of one or several acidic residues located outside of the TH8–TH9 hairpin has to occur when the T domain is already at the membrane, to ensure proper insertion and translocation. Distinguishing between these two alternatives and establishing the residues involved in pH-dependent conformational switching on the final stages of the insertion pathway will be the subject of our future studies.

Both E362Q and E349Q/D352N mutants studied here are prone to aggregation in solution under mildly acidic conditions (Figure 5). This may be the result of the mutants having a reduced negative surface charge that will otherwise prevent aggregation due to electrostatic repulsion. This is consistent with our recent hydrogen/deuterium exchange experiments in the WT protein, which identified TH9 and the loop between TH8 and TH9 as a part of a dimerization interface at low pH [34]. Thus, the negative charges of E349, D352 and E362 at neutral pH would reduce the overall propensity for aggregation, while their protonation or replacement by neutral residues would eliminate this effect. The mutant E362Q can also be less resistant to acid destabilization due to overall disruption of intramolecular contacts. According to the MD simulations performed in this study (Figure 6), the mutation E362Q disrupts the salt bridge that connects TH9 with TH1 (residues E/Q362 with R210), leading to effects on remote helices of the T domain. TH1 partially loses native contacts with TH4, making the TH1/TH4 interface less stable and easier to open through the protonation of H257 and H223 (both of them located in the same interface and responsible for triggering the initial conformational change of the protein [22,25]). This may result in an earlier exposure of the hydrophobic core of the protein and aggregation when the membrane is absent.

In conclusion, we have characterized the insertion pathways of the E362Q and E349Q/D352N mutants, capable of membrane interactions under less acidic conditions than the WT T domain. The most pronounced changes are associated with replacement of the negative charge in the middle of the TH9 helix (E362Q), which nevertheless preserves the WT-like activity. Our results indicate that the insertion pathway is modulated by multiple pH-dependent transitions and that the shift of certain steps toward less acidic pH, observed as the result of mutagenesis, does not lead to automatic shifts in the steps occurring downstream along the pathway and may result in misfolding of partially-inserted protein. We discover that conformational switching of different steps is under independent control and involves protonation of multiple titratable residues. Further studies will be directed at characterizing the complete network of protonation-driven molecular switches that trigger the membrane interactions of the T domain. A number of studies from the groups of Engelman, Reshetnyak and Andreev (see [39] and the references therein) demonstrated the utility of another pH-dependent membrane insertion system, pHLIP (pH Low Insertion Peptide), for imaging and targeting tumors. Deciphering the molecular mechanism of the T domain action is essential for the rational design of a similar molecular platform for delivering therapy to acidic diseased tissue, such as tumors [39] and ischemic myocardium [40].

## 4. Experimental Section

Materials: Palmitoyl-oleoyl-phosphatidylcholine (POPC) and palmitoyl-oleoyl-phosphatidylglycerol (POPG) were obtained from Avanti Polar Lipids (Alabaster, AL, USA). 8-aminionapthalene 1,3,6-trisulfonic acid (ANTX) and p-xylene-bis-pyridinium bromide (DPX) were obtained from Molecular Probes (Eugene, OR, USA). IANBD ester (*N*-((2-(iodoacetoxy)ethyl)-*N*-methyl)amino-7-nitrobenz-2-oxa-1,3-diazole) was purchased from Invitrogen (Carlsbad, CA, USA). The diphtheria toxin T domain (amino acids 202–378) was cloned into the Ndel- and EcoRl-treated pET15b vector containing an *N*-terminus six-His tag and a thrombin cleavage site [31]. The preparation of proteins for NBD-labeling was performed by generating the point mutation N366C with the QuickChange site-directed mutagenesis kit from Stratagene (Cedar Creek, TX, USA) and verified by DNA sequencing with T7 primer. Both of the mutants and the wild-type (WT) protein were expressed and purified as described in our earlier work [31]. The NBD-labeling was performed following a standard procedure for thiol-reactive derivatives [32,41].

LUV preparation: Large unilamellar vesicles of a diameter of 0.1 µm were prepared by extrusion [42,43] using a 1:3 mixture of POPC and POPG.

Protein Synthesis Inhibition Assays: Cytotoxicity or cell death assays were carried out using a protein synthesis inhibition assay [37]. Briefly, CHO-K1 cells (10^5^ cells per well) were intoxicated by diphtheria toxin constructs for 24 h at 37 °C, after which incorporation of L-4,5-^3^H leucine was measured. Per the NIH guidelines, these measurements were performed with a weakened strain carrying the E148S mutation in the catalytic domain to reduce the toxic potency [44].

Leakage assays: LUV with entrapped ANTS and DPX were prepared as previously described [45,46]. Briefly, previously dried lipids were resuspended in buffer containing 1 mM of ANTS and 10 mM of DPX. The lipid solutions were then frozen and thawed 20 times before extrusion. Lipid solutions were prepared at 100 mM in order to maximize loading of the solutes. To maintain the osmolarity with the external 50 mM KCl buffer, the total KCl concentration of ANTS- and DPX-containing vesicles was kept at 100 mM. Separation of the unencapsulated solutes was achieved by passing the samples through a Superose 12 1 × 30 cm.

*N*-terminus translocation assay: The ability of the T domain to translocate its *N*-terminus across the lipid bilayer was studied with thrombin-loaded LUV [23]. T domain constructs contained an *N*-terminal His-tag and a thrombin cleavage site to release the His-tag from the rest of the protein upon proteolytic treatment. Upon insertion and translocation of the purified T domain into LUV pre-loaded with thrombin, the His-tag is cleaved by the protease, resulting in a protein of lower molecular weight that can be detected by following its electrophoretic mobility. LUVs containing ~0.02 units of bovine thrombin (Fisher Bioreagents, Fair Lawn, NJ, USA) were prepared by the conventional extrusion method [42,43]. Non-encapsulated thrombin was removed by gel filtration on a Superose 12 column. In this study, 100 nM T domain was mixed with 2 mM vesicles in a total volume of 40 μL. To prevent cleavage due to vesicle rupture (rather than translocation of the terminus), 0.02 units of thrombin inhibitor hirudin (Sigma, St. Louis, MO, USA) were added to the reaction mixture. After 2 h of incubation at room temperature, 10 μL of 5× SDS sample buffer were added, and samples were boiled for 5 min and analyzed by SDS PAGE 4%–15% Tris-HCl gels.

CD measurement and analysis of thermal unfolding: CD measurements were performed using an upgraded Jasco-720 spectropolarimeter (Japan Spectroscopic Company, Tokyo, Japan). Normally, 200 scans were performed from 260 nm to 190 nm in a sample containing 4 µM of protein in 50 mM phosphate buffer using a 1-mm optical path cuvette. All spectra were corrected for the background. Temperature dependencies of unfolding were monitored by measuring ellipticity at 222 nm with a 1-degree/min scan rate. The thermal unfolding was analyzed using thermodynamic equations for a reversible two-state, *N*-to-*U* unfolding transition, where *N* and *U* are the native and unfolded state of the protein, respectively. In order to obtain the transition temperature (*Tm*) and the enthalpy changes (*∆H°*), raw data were fitted applying nonlinear least-squares analysis with six fitting parameters, *Y_N_*, *m_N_*, *Y_U_*, *m_U_*, *ΔH°* and *Tm*, with the following equations [31,47]:

*Y* = (*Y_N_* + m_N_·*T*)·*X_N_* + (*Y_U_* + m_U_·*T*)·(1−*X_N_*) (1)

*X_N_* = 1/(1 + exp(−ΔH°(1−*T*/*Tm*)/RT) (2)

where *Y* is the experimentally observed CD signal at a given temperature, *Y_N_* and *Y_U_*, represent the signals of the pure *N* and *U* states at 0 K, *m_N_* and *m_U_* are the temperature-dependencies of these CD signals for the *N* and *U* states, respectively, and *X_N_* is the fraction of the native state at temperature *T*.

Size exclusion chromatography: Previously purified monomeric fractions containing 25 μM WT or mutants were incubated in 50 mM phosphate buffer at either pH 8.0 or pH 6.5. Samples were placed onto the TSK-Gel G2000 SWXL column (Tosohaas, Japan) equilibrated at the corresponding pH with an elution rate of 0.5 mL/min. BSA (68 kDa), and ovalbumin (43 kDa), cyan fluorescent protein (22 kDa) and RNAse A (14 kDa) were used to calibrate the column.

Steady-state fluorescence spectroscopy: Fluorescence was measured using an SPEX Flurolog FL 3–22 steady-state fluorescence spectrometer (Jobin Yvon, Edison, NJ, USA) equipped with double grating excitation and emission monochromators. The measurements were made at 25 °C in 2 × 10 mm cuvettes oriented perpendicular to the excitation beam. For tryptophan fluorescence measurement, excitation emission wavelength were 280 nm, and emission spectra were recorded between 290 nm and 500 nm using excitation and emission spectral slits of 2 and 4 nm, respectively. For NBD fluorescence measurements, the excitation wavelength was 470 nm, and the emission spectra were recorded between 490 nm and 700 nm using excitation and emission spectral slits of 5 nm on both monochromators. Normally, we mixed the sample (taken from a concentrated stock) with LUV maintaining the T domain and lipid concentrations at 1 µM and 1 mM, respectively, and rapid acidification was achieved by the addition of small amounts of 2.5 M acetic buffer. All spectra were recorded after 30 min of incubation to ensure the equilibrium of the sample. Correction for the background and fitting of all spectra to calculate the position of maximum emission was done as described previously [33].

Time-resolved fluorescence spectroscopy: All of the decays were measured with a time-resolved fluorescence spectrometer (FluoTime 200, PicoQuant, Berlin, Germany) using a standard time-correlated, single-photon counting scheme. Samples were excited at 440 nm by a sub-nanosecond pulsed diode laser (LDH 440, PicoQuant, Berlin, Germany) with a repetition rate of 10 MHz. Fluorescence emission was detected at 540 nm, selected by a Sciencetech model 9030 monochromator (1200 lines/mm, spectral range 350–800 nm, linear dispersion 8 nm/mm), using a PMA-182 photomultiplier (PicoQuant).

Molecular dynamics simulations: Atom coordinates of the T domain were taken from the X-ray structure of diphtheria toxin obtained in buffer at pH 7.5 (residues 206–380 from PDB ID 1F0L) [10,48]. The model of the mutant E362Q was created by replacing side-chain atoms of E362 in the X-ray structure using tleap, and hydrogen atoms were added to both wild-type (WT) and mutant using the same software [49]. All histidines were set in the neutral state (epsilon tautomeric state), while all other residues were set to their standard ionization state at pH 7.5. Simulation boxes were prepared by adding 9113 explicit water molecules with a minimum distance between the protein and the simulation box edges of 12.0 Å. The water model used was TIP3P. The simulation boxes contained approximately 30,100 atoms with initial box dimensions of 76 Å × 77 Å × 81 Å. Sodium counter-ions were added to neutralize the charge of each system (10 and 9 cations for WT and E362Q, respectively). The ff99SB-ILDN force field for proteins was used for all simulations [50]. The SHAKE algorithm was used to constrain bond lengths between all hydrogens bonded to heavy-atoms [51]. The time-step was set to 2 fs. Periodic boundary conditions were used; a cutoff radius of 10 Å and electrostatic calculations were performed using the particle mesh Ewald (PME) method [52]. Positional restraints were applied in the protein atoms, and the solvent was minimized for 200 steps of steepest descent followed by 50 steps of the conjugate gradient descent minimization method. Then, a similar minimization was carried out with positional restraints applied in the heavy atoms of the protein. The temperature was linearly increased up to *T* = 310 K over 20 ps with harmonic restraints in the protein atoms (force constant of 10.0 kcal/mol·Å^2^) and constant volume. The final snapshot was later equilibrated over 600 ps at constant pressure conditions with *P* = 1 atm and *T* = 310 K with restraints applied to the protein heavy-atoms (10.0 kcal/mol·Å^2^), followed by three consecutive 100-ps equilibration stages with gradually reduced restraints applied to the protein heavy atoms (a force constant of 0.75, 0.5 and 0.25 kcal/mol·Å^2^ was used for each stage). Final equilibration with no restraints was carried out over 10 ns and 31 ns for the wild-type and mutant, respectively. The thermostat and barostat were Langevin and Berendsen, respectively. Production simulations of the WT and E362Q mutant were performed using the GPU-accelerated PMEMD (Particle Mesh Ewald Molecular Dynamics) and Anton supercomputer, respectively [53,54]. Both production trajectories were generated using the canonical ensemble with constant number of particles, volume and temperature (NVT). In Anton simulations, the electrostatic interactions were computed by the Gaussian split Ewald method with a grid size of 64 × 64 × 64 and a cutoff radius of 14.0 Å [55]. A reversible multiple-time step algorithm was used with a time step of 6 fs for the long-range non-bonded forces and 2 fs for bonded and short-range non-bonded interactions [56]. Bonded hydrogens were constrained with the M-SHAKE algorithm [57]. The Berendsen thermostat and barostat were set to *T* = 310 K (tau = 1 ps) and a pressure of 1 atm (tau = 2 ps), respectively. System coordinates were saved every 60 ps. Production MD simulations of WT and E362Q mutant were performed over 3630 ns and 3481 ns, respectively. Trajectory analysis was carried out using the program ptraj available in Amber12 (University of California, San Francisco, CA, USA, 2012) [49], and molecular figures were created using VMD 1.9.1 (University of Illinois at Urbana-Champaign, Urbana, IL, USA, 2012) [58].

## Supplementary Materials

Supplementary materials can be accessed at: http://www.mdpi.com/2072-6651/7/4/1303/s1.
