# Supplementary material for: Role of Acidic Residues in Helices TH8–TH9 in Membrane Interactions of the Diphtheria Toxin T Domain
Source: Toxins (Basel). 2015 Apr 14;7(4):1303–23. doi: 10.3390/toxins7041303 (PMC4417968; doi:10.3390/toxins7041303)
Supplement: Supplementary file 1 [file toxins-07-01303-s001.pdf]

Supplementary Information

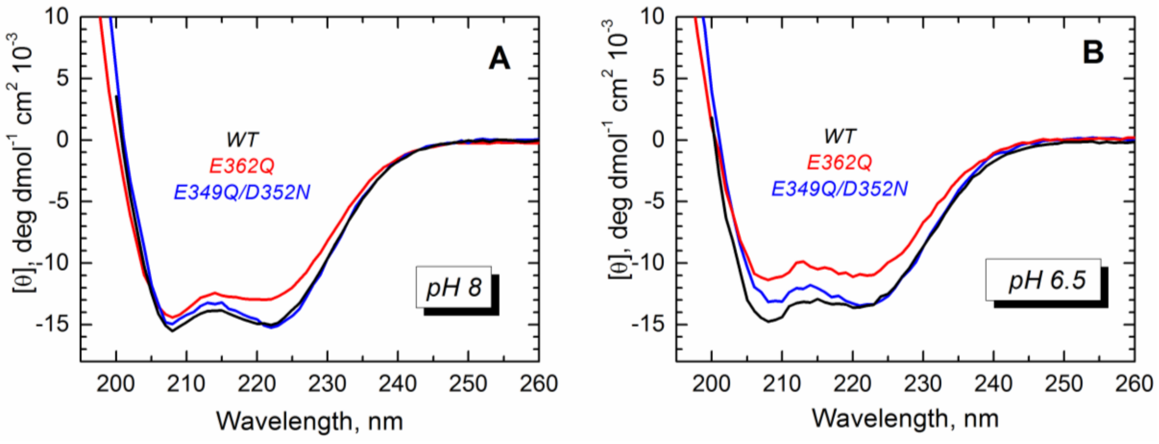

Figure S1. CD spectra of the T domain WT and mutants in solution at (**A**) pH 8.0 and (**B**) pH 6.5 show that the mutant E362Q (red) is slightly unfolded as compared to the WT, while the double mutant E349Q/D352N (blue) is just marginally different from the WT.

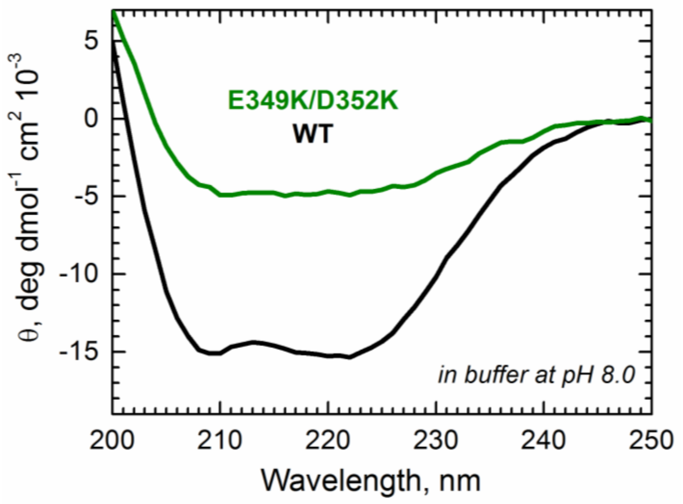

Figure S2. CD spectra of the T domain WT (black) and double mutant E349K/D352K (olive) in buffer at pH 8.0. Results show the loss of molar ellipticity upon replacement of resides E349 and D352 with lysines, suggesting major misfolding of the protein in solution. Data were collected as described in the Experimental Section.

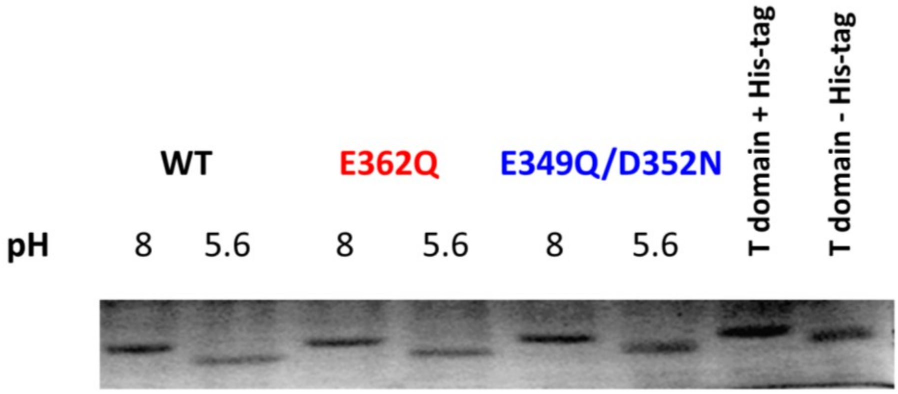

Figure S3. Example of SDS-PAGE data used for the measurements of the translocation activity of the T domain. The details of the methodology are described in Rodnin *et al.*, 2014 [36]. Briefly, the T domain of WT or mutant was mixed with LUV pre-loaded with thrombin. Upon addition of acid to reach the indicated pH, the samples were incubated for 2 h at room temperature. Translocation of the N-terminus was judged on the basis of the cleavage of the N-terminus His-tag attached through a thrombin site, which changes the electrophoretic mobility of the protein. The last two lanes of the SDS-PAGE are reference reactions in the absence of LUV to show the differences in electrophoretic mobility upon thrombin cleavage. The data show that both mutants retain translocation activity.
